# Outcome of infection with omicron SARS‐CoV‐2 variant in patients with hematological malignancies: An EPICOVIDEHA survey report

**DOI:** 10.1002/ajh.26626

**Published:** 2022-06-28

**Authors:** Ola Blennow, Jon Salmanton‐García, Piotr Nowak, Federico Itri, Jaap Van Doesum, Alberto López‐García, Francesca Farina, Ozren Jaksic, László Imre Pinczés, Yavuz M. Bilgin, Iker Falces‐Romero, Moraima Jiménez, Irati Ormazabal‐Vélez, Barbora Weinbergerová, Rémy Duléry, Zlate Stojanoski, Tobias Lahmer, Noemí Fernández, José‐Ángel Hernández‐Rivas, Verena Petzer, Nick De Jonge, Andreas Glenthøj, Cristina De Ramón, Monika M. Biernat, Nicola Fracchiolla, Avinash Aujayeb, Jens Van Praet, Martin Schönlein, Gustavo‐Adolfo Méndez, Chiara Cattaneo, Anna Guidetti, Mariarita Sciumè, Emanuele Ammatuna, Raul Cordoba, Nicole García‐Poutón, Stefanie Gräfe, Alba Cabirta, Dominik Wolf, Anna Nordlander, Ramón García‐Sanz, Mario Delia, Caroline Berg Venemyr, Clara Brones, Roberta Di Blasi, Elizabeth De Kort, Stef Meers, Sylvain Lamure, Laura Serrano, Maria Merelli, Nicola Coppola, Rui Bergantim, Caroline Besson, Milena Kohn, Jessica Petiti, Carolina Garcia‐Vidal, Michelina Dargenio, François Danion, Marina Machado, Rebeca Bailén‐Almorox, Martin Hoenigl, Giulia Dragonetti, Louis Yi Ann Chai, Chi Shan Kho, Matteo Bonanni, Raphaël Liévin, Francesco Marchesi, Oliver A. Cornely, Livio Pagano

**Affiliations:** ^1^ Department of Infectious Diseases Karolinska University Hospital Stockholm Sweden; ^2^ Faculty of Medicine and University Hospital Cologne, Department I of Internal Medicine, Excellence Center for Medical Mycology (ECMM) University of Cologne Cologne Germany; ^3^ Faculty of Medicine and University Hospital Cologne, Cologne Excellence Cluster on Cellular Stress Responses in Aging‐Associated Diseases (CECAD) University of Cologne Cologne Germany; ^4^ Department of Clinical and Biological Sciences, San Luigi Gonzaga Hospital – Orbassano Orbassano Italy; ^5^ Department of Hematology, University Medical Center Groningen Groningen The Netherlands; ^6^ Health Research Institute IIS‐FJD Fundacion Jimenez Diaz University Hospital Madrid Spain; ^7^ U.O. Ematologia e Trapianto Midollo, Dipartimento di Oncologia Istituto Scientifico San Raffaele Milan Italy; ^8^ Department of Hematology, University Hospital Dubrava Zagreb Croatia; ^9^ Division of Hematology, Department of Internal Medicine, Faculty of Medicine University of Debrecen Debrecen Hungary; ^10^ Department of Internal Medicine/Hematology, ADRZ Goes Netherlands; ^11^ Microbiology and Parasitology Department University Hospital La Paz Madrid Spain; ^12^ CIBERINFEC Instituto de Salud Carlos III Madrid Spain; ^13^ Department of Hematology, Vall d'Hebron Hospital Universitari, Experimental Hematology, Vall d'Hebron Institute of Oncology (VHIO) Vall d'Hebron Barcelona Hospital Campus Barcelona Spain; ^14^ Departament de Medicina Universitat Autònoma de Barcelona Bellaterra Spain; ^15^ Department of Haematology, Complejo Hospitalario de Navarra Pamplona Spain; ^16^ Department of Internal Medicine, Hematology and Oncology Masaryk University and University Hospital Brno Brno Czech Republic; ^17^ INSERM UMRs 938, Service d'Hématologie Clinique et de Thérapie Cellulaire, Hôpital Saint Antoine, AP‐HP Sorbonne Université Paris France; ^18^ University Clinic for Hematology, Medical Faculty, Ss Cyril and Methodius University of Skopje Skopje North Macedonia; ^19^ Medizinische Klinik II, Klinikum rechts der Isar TU München Munich Germany; ^20^ Department of Hematology, Hospital Universitario Marqués de Valdecilla Santander Spain; ^21^ Deparment of Hematology, Hospital Universitario Infanta Leonor Madrid Spain; ^22^ Department of Hematology and Oncology Medical University of Innsbruck Innsbruck Austria; ^23^ Department of Hematology, Cancer Center Amsterdam Amsterdam UMC, Vrije Universiteit Amsterdam Amsterdam The Netherlands; ^24^ Department of Hematology Copenhagen University Hospital Rigshospitalet Copenhagen Denmark; ^25^ Hematology Department Hospital Universitario de Salamanca Salamanca Spain; ^26^ Centro de Investigación del Cáncer‐IBMCC (USAL‐CSIC) IBSAL Salamanca Spain; ^27^ Department of Haematology, Blood Neoplasms, and Bone Marrow Transplantation Wroclaw Medical University Wroclaw Poland; ^28^ Hematology Unit Fondazione IRCCS Ca' Granda Ospedale Maggiore Policlinico Milan Italy; ^29^ Respiratory Department, Northumbria Healthcare Newcastle UK; ^30^ Department of Nephrology and Infectious diseases AZ Sint‐Jan Brugge‐Oostende AV Brugge Belgium; ^31^ Department of Oncology, Hematology and Bone Marrow Transplantation with Section of Pneumology University Medical Center Hamburg‐Eppendorf Hamburg Germany; ^32^ Servicio de Infectología y Control de Infecciones Hospital Escuela de Agudos Dr. Ramón Madariaga Posadas Misiones Argentina; ^33^ Hematology Unit ASST‐Spedali Civili Brescia Italy; ^34^ Division of Hematology and Bone Marrow Transplantation, Fondazione IRCCS Istituto Nazionale dei Tumori Milan Italy; ^35^ Department of Infectious Diseases, Hospital Clinic de Barcelona University of Barcelona, IDIBAPS Barcelona Spain; ^36^ Department of Oncology, Hematology, and Bone Marrow Transplantation with Section of Pneumology, Universitätsklinikum Hamburg Eppendorf Hamburg Germany; ^37^ Hematology and Stem Cell Transplantation Unit AOUC Policlinico Bari Italy; ^38^ Service d'Hématologie, Centre Hospitalier de Versailles Le Chesnay France; ^39^ Hemato‐Oncology Department, Hopital Saint Louis Paris France; ^40^ Department of Hematology Radboud University Medical Center Nijmegen The Netherlands; ^41^ Department Oncology, AZ KLINA Brasschaat Belgium; ^42^ Department of Clinical Hematology, Montpellier University Hospital, IGMM UMR5535 CNRS, University of Montpellier Montpellier France; ^43^ Deparment of Hematology, Hospital Universitario de Cabueñes Gijón Spain; ^44^ Infectious Diseas Clinic ASU FC Udine Hospital Udine Italy; ^45^ Department of Mental Health and Public Medicine Universitry of Campania Naples Italy; ^46^ Department of Hematology, Centro Hospitalar e Universitário de São João Porto Portugal; ^47^ Department of Clinical and Biological Sciences University of Turin Turin Italy; ^48^ Hematology and Stem Cell Transplant Unit, "Vito Fazzi" Hospital Lecce Italy; ^49^ Department of Infectious Diseases CHU de Strasbourg Strasbourg France; ^50^ Clinical Microbiology and Infectious Diseases Department Hospital General Universitario Gregorio Marañón Madrid Spain; ^51^ Department of Hematology and Hemotherapy Hospital General Universitario Gregorio Marañón Madrid Spain; ^52^ Division of Infectious Diseases and Global Public Health, Department of Medicine University of California San Diego San Diego California USA; ^53^ Clinical and Translational Fungal‐Working Group University of California San Diego La Jolla California USA; ^54^ Division of Infectious Diseases, Department of Internal Medicine Medical University of Graz Graz Austria; ^55^ Hematology Unit Fondazione Policlinico Universitario Agostino Gemelli – IRCCS Rome Italy; ^56^ National University Health System, University Medicine Center, Division of Infectious Diseases Singapore; ^57^ Pamela Youde Nethersole Eastern Hospital Hong Kong Hong Kong SAR; ^58^ Hematology Unit Università Cattolica del Sacro Cuore Rome Italy; ^59^ Hematology and Stem Cell Transplant Unit IRCCS Regina Elena National Cancer Institute Rome Italy; ^60^ Faculty of Medicine and University Hospital Cologne, Clinical Trials Centre Cologne (ZKS Köln) University of Cologne Cologne Germany; ^61^ Faculty of Medicine and University Hospital Cologne, Center for Molecular Medicine Cologne (CMMC) University of Cologne Cologne Germany; ^62^ German Centre for Infection Research (DZIF), Partner Site Bonn‐Cologne Cologne Germany

To the Editor:

Severe acute respiratory syndrome coronavirus 2 (SARS‐CoV‐2) infection has caused high mortality in patients with hematological malignancies (HM).^1^ The newly emerged omicron variants of SARS‐CoV‐2 harbor multiple novel spike protein mutations that raise concerns about vaccine efficiency and antiviral efficacy of the available therapeutic monoclonal antibodies.^2^ The first published clinical data in immunocompetent patients have found that infection with omicron variants is associated with reduced vaccine efficiency compared to the delta variants, but decreased hospital admission and mortality.^3^, ^4^ Preliminary, prepublished, data from a large case–control study have shown that the vaccine effect against omicron in immunocompromised patients, including HM patients, is even more reduced, but data regarding clinical outcomes are lacking.^5^ The aim of this study was to describe risk factors, antiviral treatment and outcomes of SARS‐CoV‐2 omicron variant infection in 593 HM patients included in the EPICOVIDEHA registry.

EPICOVIDEHA is an international open web‐based registry for patients with HM infected with SARS‐CoV‐2.^1^, ^6^ Both hospitalized and nonhospitalized patients are eligible for inclusion. The questionnaire includes data on the HM, SARS‐CoV‐2 vaccination status, risk factors for severe COVID‐19 infection, SARS‐CoV‐2 virus variant, antiviral treatment, and outcomes including mortality (eFigure 1 and eTable 4). Classification of attributable, contributable, or nonattributable death is made by the reporting physician. All included cases are validated by experts with previous experience in research studies of hematological malignancies and infectious diseases at the University Hospital Cologne, Cologne, Germany.

Critical infection was defined as admittance to an intermediate and/or intensive care unit. Independent predictors for mortality were assed via a Cox proportional hazard model. Risk factors for critical SARS‐CoV‐2 infection were determined with a logistic regression. Variables with a p‐value ≤.1 in the univariable models were considered for multivariable analysis. Multivariable regression models (both Cox and logistic) were calculated with the Wald backward method, and only those variables that were statistically significant displayed. Log‐rank test was used to compare the survival probability of the patients included in the different models. A priori sample size calculation was not applied in this exploratory study. SPSSv25.0 was employed for statistical analyses (SPSS, IBM Corp.).

In total 593 HM patients infected with omicron were included, whereof 309 patients were admitted to hospital and 284 patients stayed home (eTable 1). Hospitalized patients were older than nonhospitalized patients, had more comorbidities, and had a higher proportion of patients with neutropenia, lymphocytopenia, active hematological malignancy, and treatment with anti‐CD20 antibodies (eTable 1). At least one dose of vaccine had been administered to 83.1% of all patients, with a nonsignificant difference between nonhospitalized and hospitalized patients, 86.3% compared to 80.3% (p = .157) (eTable 1, eTable 2).

Overall mortality among hospitalized patients was 16.5% (51/309), of which 61% was classified as attributable to omicron, 35.3% contributable, and 3.9% unrelated. Factors associated with attributable and contributable mortality in hospitalized patients were older age (analyzed as continuous variable, hazard ratio (HR) 1.05 (95% confidence interval (CI) 1.02–1.07, p < .001]) and active malignancy (HR 2.5 [95% CI 1.3–4.8, p = .007]) (Table 1). Having received at least one dose of SARS‐CoV‐2 vaccine was protective in univariable analysis (HR 0.53 [95% CI 0.29–0.96, p = .036]), but did not reach statistical significance in multivariable analysis (HR 0.58 [95% CI 0.32–1.05, p = .074]) (eFigure 2a, Table 1).

**TABLE 1:** Risk factor analysis for omicron‐related mortality in hospitalized patients

	Hospital															
	Overall								Critical							
	Univariable				Multivariable				Univariable				Multivariable			
	p value	HR	95% CI		p value	HR	95% CI		p value	HR	95% CI		p value	HR	95% CI	
			Lower	Upper			Lower	Upper			Lower	Upper			Lower	Upper
**Sex**																
Female	—	—	—	—					—	—	—	—				
Male	.609	0.866	0.500	1.501					.800	0.894	0.377	2.124				
**Age**	<.001	1.047	1.022	1.072	<0.001	1.044	1.020	1.068	0.134	1.029	0.991	1.069				
**Status of malignancy at COVID‐19 onset** ^a^																
Controlled malignancy	—	—	—	—	—	—	—	—	—	—	—	—	—	—	—	—
Stable malignancy	0.348	1.526	0.632	3.684	0.668	1.215	0.499	2.957	0.014	4.780	1.370	16.676	0.003	8.230	2.026	33.439
Active malignancy	0.003	2.726	1.421	5.229	0.007	2.473	1.284	4.762	0.088	2.511	0.871	7.240	0.055	2.887	0.977	8.529
**Baseline malignancy**																
Lymphoproliferative malignancies	—	—	—	—					—	—	—	—				
Myeloproliferative malignancies	0.508	0.798	0.409	1.556					0.758	1.153	0.465	2.862				
Aplastic anemia	0.974	0.000	0.000													
**Previous SARS‐CoV‐2 vaccination**	0.036	0.530	0.293	0.959	0.074	0.581	0.320	1.054	0.592	0.780	0.314	1.934				
**COVID‐19 treatment** ^b^																
No treatment	—	—	—	—					—	—	—	—	—	—	—	—
Any remdesivir, minus sotrovimab	0.857	0.941	0.487	1.817					0.953	0.967	0.314	2.975	0.200	0.452	0.134	1.521
Any sotrovimab + any tixagevimab/cilgavimab	0.134	0.531	0.232	1.216					0.021	0.172	0.039	0.766	0.010	0.134	0.029	0.614
Plasma only + molnupiravir only	0.639	1.330	0.404	4.377					0.776	1.242	0.280	5.513	0.604	1.491	0.330	6.738
**Previous administration of antiCD20**	0.760	1.090	0.626	1.898					0.187	0.528	0.205	1.363				

Abbreviations: 95% CI, 95% confidence interval; COVID‐19, coronavirus disease 2019, HR, hazard ratio; SARS‐CoV‐2, severe acute respiratory syndrome coronavirus 2.

^a^ Controlled malignancy: Complete remission or partial remission; active malignancy: onset or refractory/resistant.

^b^ No treatment: Includes treatment with acyclovir, favipiravir, casivirimab/imdevimab, bamlanvimab/etesivimab and regdanvimab; Any remdesivir, minus sotrovimab: Includes all antiviral treatment which included remdesivir except for sotrovimab and tixagevimab/cilgavimab; Any sotrovimab + any tixagevimab/cilgavimab: Includes all antiviral treatment which included sotrovimab or tixagevimab/cilgavimab; Plasma only + molnupiravir only: Includes single therapy with convalescent plasma or molnupiravir.

Progression to critical infection occurred in 53 (17.0%) of hospitalized patients. Risk factor for progression to critical COVID‐19 was pre‐existent chronic pulmonary disease (odds ratio (OR) 3.2 [95% CI 1.4–7.3, p = .005]) (eTable 3). Baseline lymphocytes of ≥500 cells/mm^3^ were protective (OR 0.4 [95% CI 0.18–0.90, p = .027]) while a lymphocyte count between 200 and 499 cells/mm^3^ was protective in uni‐ but not multivariable analysis (OR 0.44 [95% CI 0.16–1.20, p = .108]). Three doses of vaccine were protective (OR 0.29 [95% CI 0.13–0.64, p = .003]), but not two doses (OR 0.73 [95% CI 0.33–1.66, p = .457] (eTable 3). Mortality among patients with critical infection was 39.2% (20/53). Administration of antibody‐based antiviral treatment with sotrovimab or tixagevimab/cilgavimab was associated with a lower risk for mortality in critical infection (HR 0.13, [95% CI 0.02–0.61, p = .010]) (eFigure 2b, Table 1), while administration of other SARS‐CoV‐2 directed monoclonal antibodies was not (data not shown).

This observational study from the EPICOVIDEHA registry is the first report on clinical data in a large cohort of omicron‐infected HM patients. The main finding is that infection with omicron is associated with considerable attributable mortality in HM patients. Additionally, we found factors associated with a potential antibody response, that is, not having severe lymphocytopenia and having received at least three doses of SARS‐CoV‐2 vaccine, and treatment with monoclonal antibodies with in vitro effect against omicron, to be protective against progression to critical infection and death.

The mortality among hospitalized HM patients was 16.5% which is lower than during the COVID‐19 waves of 2020 and 2021, but considerable higher than previously reported mortality rates in immunocompetent patients with omicron infection.^1^, ^3^, ^4^ Data regarding outcomes in HM patients with omicron are scarce, but our finding is in agreement with a small recent preliminary report on omicron in chronic lymphoid leukemia patients, where 23% 30‐day mortality was reported.^7^ Thus, as opposed to immunocompetent patients, infection with omicron in HM patients is still associated with a considerable mortality in hospitalized patients.

Due to serological data not being registered consistently by all participating centers, the protective effect of vaccination was analyzed according to number of doses administrated. For the whole cohort, the vaccination rate was numerically higher in non‐hospitalized patients than hospitalized patients, 86.3% compared to 80.3%, respectively. Administration of at least one dose of vaccine was protective against death in all hospitalized patients in univariable analysis but not in multivariable analysis (p = .074). Three doses of vaccine were protective against progression to critical infection in hospitalized patients, while two doses were not, a finding that is well in line with the additional booster effect against omicron in immunocompetent patients.^3^, ^8^ Interestingly, lymphocytopenia, which has been associated with a poor vaccine response, was also associated with progression to critical infection.^9^ Finally, among patients that progressed to critical infection, vaccination was not associated with a protective effect against death, contrary to treatment with monoclonal antibodies with in vitro effect against omicron.^2^ These findings raise the hypothesis that while vaccination appears to be protective against severe infection and death, lack of response, as manifested by progression to critical infection despite vaccination, may be at least in part compensated by passive immunization using SARS‐CoV‐2‐antagonizing monoclonal antibodies. This hypothesis is in line with the findings from a large, randomized treatment study, reporting significantly decreased mortality with administration of monoclonal antibodies in hospitalized seronegative immunocompetent patients.^10^

Important limitations of our study include the retrospective observational design and the accompanying risk for selection bias at participating sites, lack of serological data, and lack of sequencing data which would enable distinction between the different omicron variants. Due to these limitations, caution must be taken in interpretation and generalization of the results.

In conclusion, infection with omicron in patients with HM was associated with considerable morbidity and mortality, vaccination with at least three doses was protective against progression to critical infection, and treatment with monoclonal antibodies was associated with reduced mortality in patients that had progressed to critical infection.

## FUNDING INFORMATION

EPICOVIDEHA has received funds from Optics COMMIT (COVID‐19 Unmet Medical Needs and Associated Research Extension) COVID‐19 RFP program by GILEAD Science, United States (Project 2020‐8223).

## CONFLICT OF INTEREST

The authors declare that they have no competing interests for this work.

## Supporting information


**Appendix S1** Supplementary Information

## Data Availability

The datasets used and/or analyzed during the current study are not publicly available due to individual privacy reason, but are available from the corresponding author on reasonable request.
