# Supplementary material for: Outcome of infection with omicron SARS‐CoV‐2 variant in patients with hematological malignancies: An EPICOVIDEHA survey report
Source: Am J Hematol. 2022 Jun 28;97(8):E312–7. doi: 10.1002/ajh.26626 (PMC9349555; doi:10.1002/ajh.26626)
Supplement: Supplementary file 1 — Appendix S1 Supplementary Information [file AJH-97-E312-s001.docx]

## Outcome of infection with omicron SARS-CoV-2 variant in patients with hematological malignancies-An EPICOVIDEHA survey report On-line only supplement

**eFigure 1.** Included patients according to country. Spain (n=150), Italy (n=137), Netherlands (n=89), France (n=31), Croatia and Hungary (n=30, each), Germany (n=21), Czech Republic (n=17), North Macedonia (n=14), Austria (n=13), Belgium (n=11), Denmark, Poland and Sweden (n=10, each), United Kingdom (n=9), Argentina (n=7), Portugal (n=2), and Hong Kong SAR and Singapore (n=1, each).


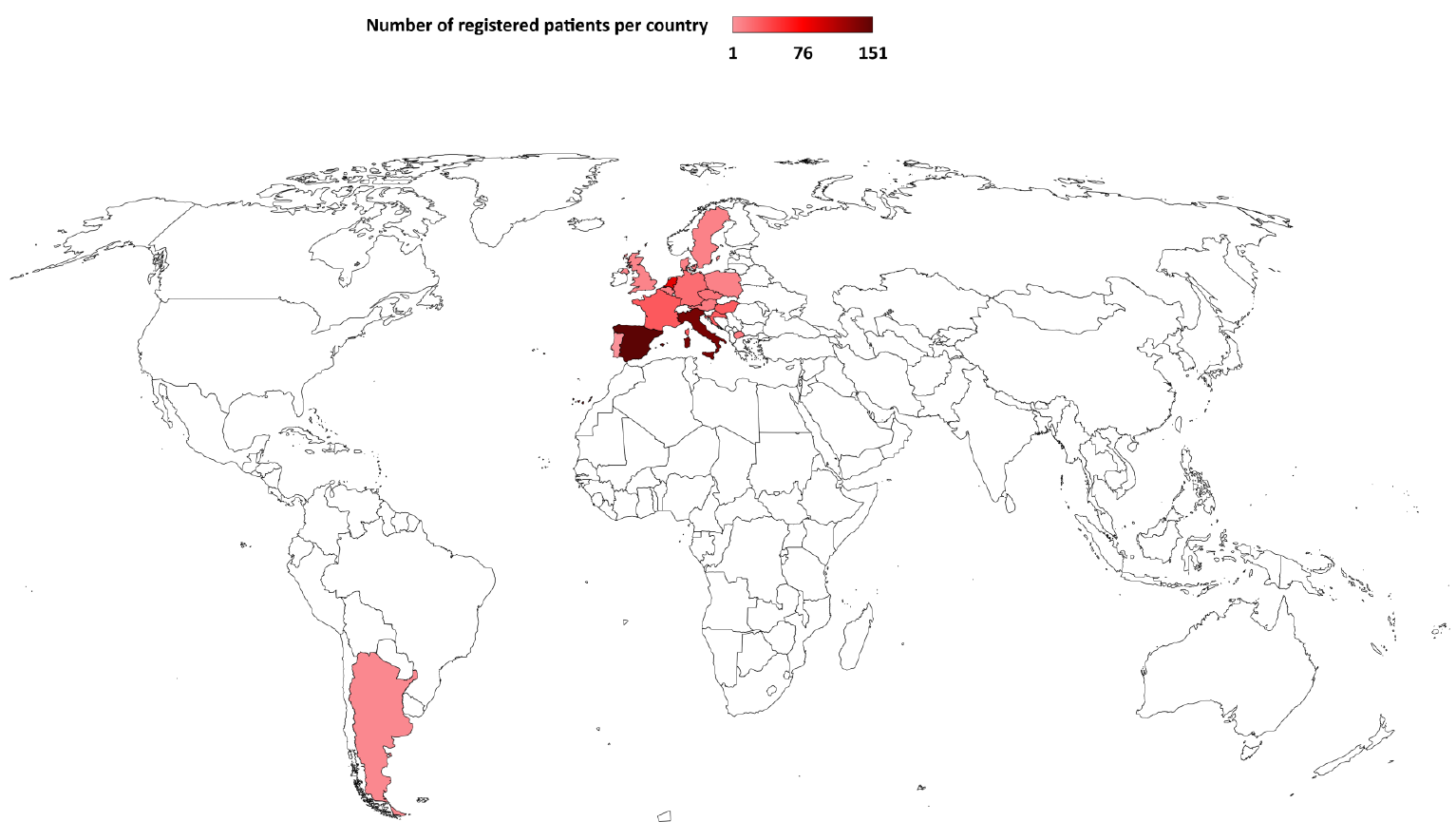


**eFigure 2.** Kaplan-Meier plot for COVID-19 associated mortality in hospitalized patients.

***(A) All hospitalized patients.***


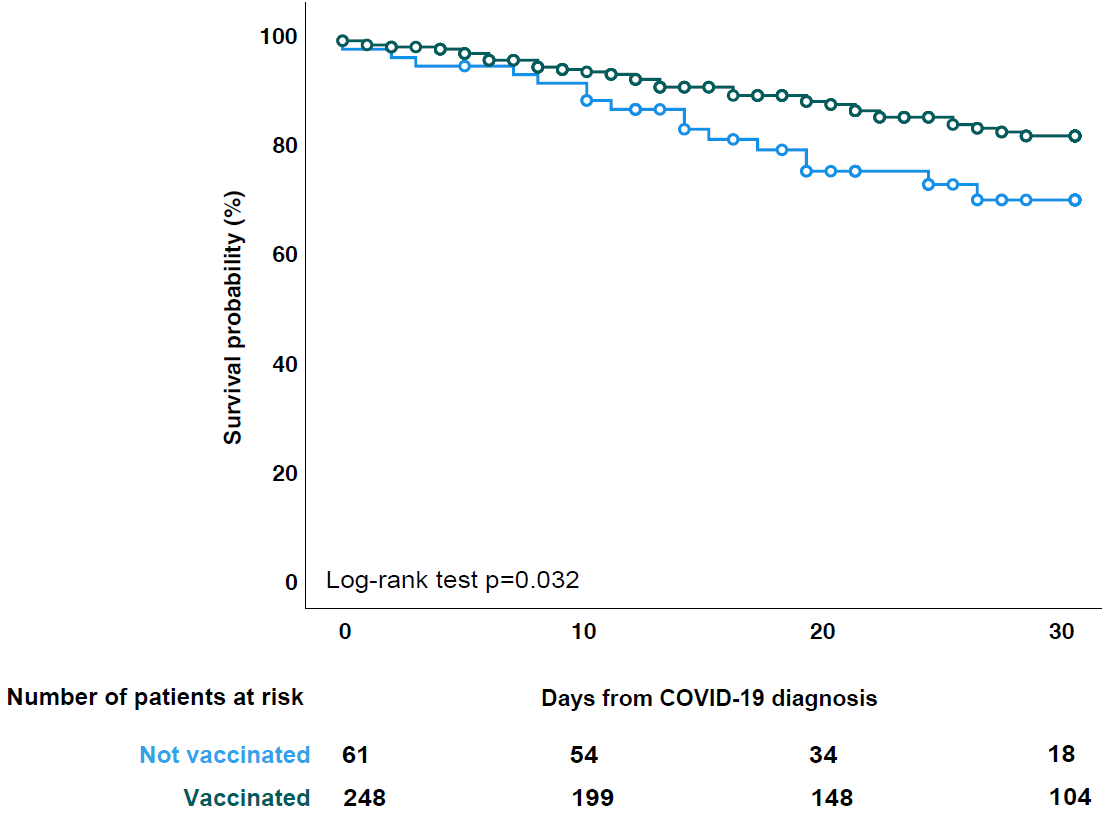


***(B) Hospitalized patients with critical infection.*** *3 patients receiving treatment with convalescent plasma or molnupiravir not included because of low numbers.* “*No treatment” also includes treatment with acyclovir, favipiravir, casivirimab/imdevimab, bamlanvimab/etesivimab and regdanvimab. “Any remdesivir, minus sotrovimab” includes all antiviral treatments which included remdesivir except for sotrovimab and tixagevimab/cilgavimab. “Any sotrovimab + any tixagevimab/cilgavimab” includes all antiviral treatment which included sotrovimab or tixagevimab/cilgavimab*


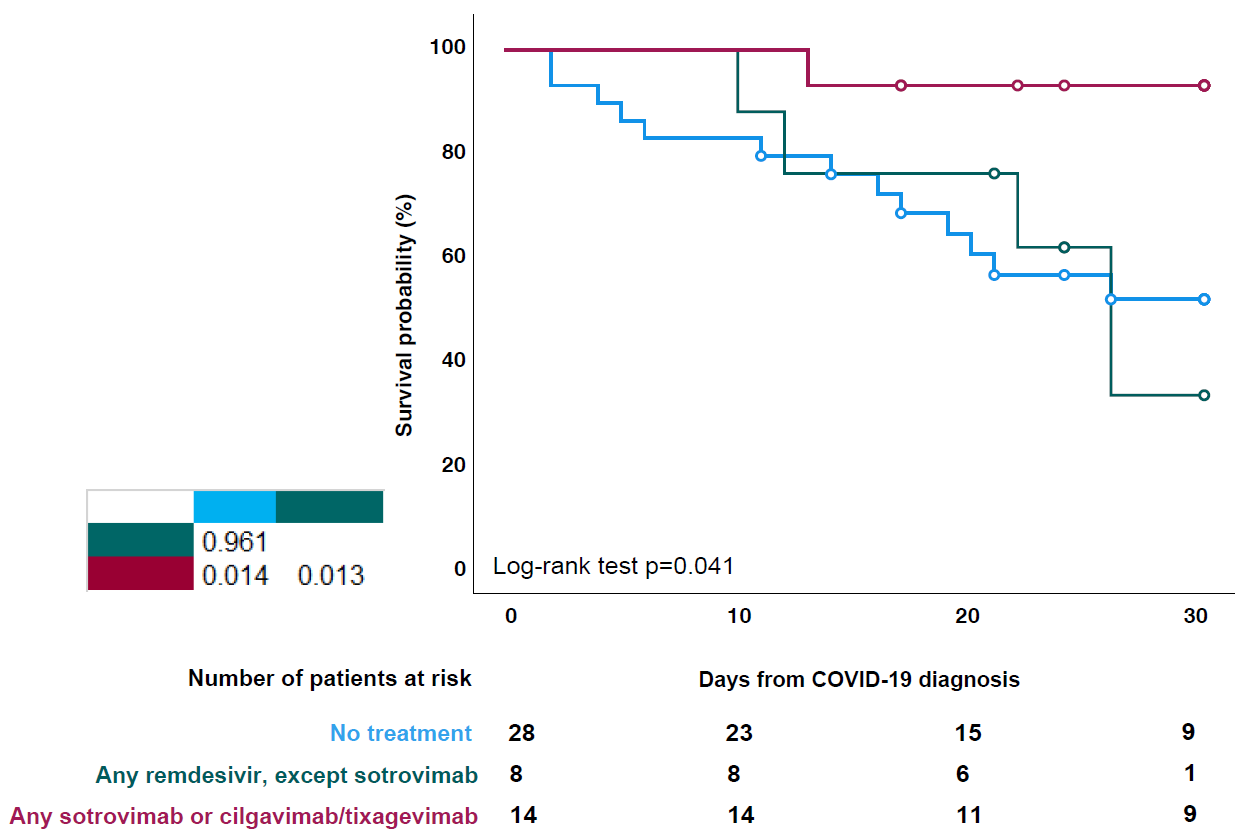
**eTable 1.** Characteristics and outcome data for 593 hospitalized and non-hospitalized patients

|  | **Overall** *(n=593)* | | **Home** *(n=284)* | | **Hospital** | | | | **p value** |
| --- | --- | --- | --- | --- | --- | --- | --- | --- | --- |
|  |  |  |  |  | ***Overall*** (n=309) | | ***Critical infection*** (n=53) | |  |
|  | **n** | **%** | **n** | **%** | **n** | **%** | **n** | **%** | *Home vs Hospital* |
| **Sex** |  |  |  |  |  |  |  |  |  |
| Female | 258 | 43.5% | 124 | 48.1% | 134 | 51.9% | 23 | 17.2% | 0.942 |
| Male | 335 | 56.5% | 160 | 47.8% | 175 | 52.2% | 30 | 17.1% |  |
| **Age***, (IQR) [absolute range]* | 66 (54-75) [18-97] | | 64 (51-74) [20-94] | | 68 (59-77) [18-97] | | 65 (54-71) [29-88] | | <0.001 |
| 18-25 years old | 20 | 3.4% | 14 | 70.0% | 6 | 30.0% | 0 | 0.0% | 0.002 |
| 26-50 years old | 93 | 15.7% | 56 | 60.2% | 37 | 39.8% | 6 | 16.2% |  |
| 51-69 years old | 236 | 39.8% | 115 | 48.7% | 121 | 51.3% | 28 | 23.1% |  |
| ≥ 70 years old | 244 | 41.1% | 99 | 40.6% | 145 | 59.4% | 19 | 13.1% |  |
| **Comorbidities before COVID-19** ^a^ |  |  |  |  |  |  |  |  |  |
| No comorbidities | 234 | 39.5% | 132 | 56.4% | 102 | 43.6% | 16 | 15.7% | <0.001 |
| 1 comorbidity | 199 | 33.6% | 95 | 47.7% | 104 | 52.3% | 19 | 18.3% |  |
| 2 comorbidities | 99 | 16.7% | 34 | 34.3% | 65 | 65.7% | 11 | 16.9% |  |
| 3 or more comorbidities | 61 | 10.3% | 23 | 37.7% | 38 | 62.3% | 7 | 18.4% |  |
| **Baseline malignancy** |  |  |  |  |  |  |  |  | 0.620 |
| Lymphoproliferative malignancies | 417 | 70.3% | 195 | 46.8% | 222 | 53.2% | 34 | 15.3% |  |
| Myeloproliferative malignancies | 154 | 26.0% | 74 | 48.1% | 80 | 51.9% | 18 | 22.5% |  |
| Other malignancies | 22 | 3.7% | 15 | 68.2% | 7 | 31.8% | 1 | 14.3% |  |
| **Status of malignancy at COVID-19 onset** ^b^ |  |  |  |  |  |  |  |  | <0.001 |
| Controlled malignancy | 320 | 54.0% | 187 | 58.4% | 133 | 41.6% | 21 | 15.8% |  |
| Stable malignancy | 104 | 17.5% | 45 | 43.3% | 59 | 56.7% | 9 | 15.3% |  |
| Active malignancy | 168 | 28.3% | 52 | 31.0% | 116 | 69.0% | 23 | 19.8% |  |
| **Malignancy treatment before COVID-19** | 548 | 92.4% | 267 | 48.7% | 281 | 51.3% | 50 | 17.8% | 0.157 |
| **Previous administration of anti-CD20 before COVID-19** | 208 | 35.1% | 85 | 40.9% | 123 | 59.1% | 18 | 14.6% | 0.012 |
| **Neutropenia** ^c^ | 38 | 6.4% | 11 | 28.9% | 27 | 71.1% | 5 | 18.5% | 0.048 |
| **Lymphopenia** ^d^ | 48 | 8.1% | 8 | 16.7% | 40 | 83.3% | 12 | 30.0% | <0.001 |
| **Previous SARS-CoV-2 vaccination** | 493 | 83.1% | 245 | 49.7% | 248 | 50.3% | 37 | 14.9% | 0.051 |
| Not vaccinated | 100 | 16.9% | 39 | 39.0% | 61 | 61.0% | 16 | 26.2% | 0.030 |
| One dose | 27 | 4.6% | 11 | 40.7% | 16 | 59.3% | 1 | 6.3% |  |
| >One dose | 466 | 78.6% | 234 | 50.2% | 232 | 49.8% | 36 | 15.5% |  |
| **COVID-19** **treatment** ^e^ |  |  |  |  |  |  |  |  | <0.001 |
| No treatment | 376 | 63.4% | 224 | 59.6% | 152 | 40.4% | 28 | 18.4% |  |
| Any remdesivir, minus sotrovimab | 80 | 13.5% | 7 | 8.8% | 73 | 91.3% | 8 | 11.0% |  |
| Any sotrovimab + any tixagevimab/cilgavimab | 114 | 19.2% | 40 | 35.1% | 74 | 64.9% | 14 | 18.9% |  |
| Plasma only + molnupiravir only | 22 | 3.7% | 12 | 54.5% | 10 | 45.5% | 3 | 30.0% |  |
| **Stay during COVID**-19 |  |  |  |  |  |  |  |  |  |
| Duration of the stay in hospital*, (IQR) [absolute range]* | 10 (6-19) [1-135] | |  | | 10 (6-18) [1-135] | | 17 (10-30) [1-60] | | - |
| Intermediate care | 16 | 2.7% |  |  | 16 | 100.0% | 16 | 100.0% | - |
| *Duration of the stay in intermediate care, (IQR) [absolute range]* | 10 (5-21) [1-44] | |  | | 10 (5-24) [1-44] | | 10 (5-24) [1-44] | | - |
| ICU | 44 | 7.4% |  |  | 44 | 100.0% | 44 | 100.0% | - |
| *Duration of the stay in ICU, (IQR) [absolute range]* | 12 (6-15.5) [1-30] | |  | | 11 (6-15) [1-30] | | 11 (6-15) [1-30] | | - |
| *Invasive mechanical ventilation* | 18 | 3.0% |  |  | 18 | 100.0% | 18 | 100.0% | - |
| *Non-invasive mechanical ventilation* | 18 | 3.0% |  |  | 18 | 100.0% | 18 | 100.0% | - |
| **Outcome** |  |  |  |  |  |  |  |  |  |
| *Observation time, (IQR) [absolute range]* | 23 (12-30) [0-30] | | 23 (10.5-30) [0-30] | | 23 (12-30) [0-30] | | 24 (16-30) [2-30] | | 0.375 |
| Mortality | 54 | 9.1% | 3 | 5.6% | 51 | 94.4% | 20 | 39.2% | <0.001 |
| *Reason for death* |  |  |  |  |  |  |  |  |  |
| COVID-19 | 32 | 5.4% | 1 | 3.1% | 31 | 96.9% | 13 | 41.9% | 0.199 |
| COVID-19 + hematological malignancy | 19 | 3.2% | 1 | 5.3% | 18 | 94.7% | 6 | 33.3% |  |
| Hematological malignancies +/- other reasons | 3 | 0.5% | 1 | 33.3% | 2 | 66.7% | 1 | 50.0% |  |

COVID-19, coronavirus disease 2019; ICU, intensive care unit; IQR, interquartile range; SARS-CoV-2, severe acute respiratory syndrome coronavirus 2

^a^Includes chronic cardiopathy, chronic pulmonary disease, liver disease, diabetes mellitus, obesity (BMI >30, renal impairment (creatinine >2 mg/dl), and history of smoking

^b^Controlled malignancy: Complete remission or partial remission; active malignancy: Onset or refractory/resistant

^c^Absolute neutrophil count <500 cells/mm3

^d^<200 cells/mm3

^e^No treatment: Includes treatment with acyclovir, favipiravir, casivirimab/imdevimab, bamlanvimab/etesivimab and regdanvimab; Any remdesivir, minus sotrovimab: Includes all antiviral treatment which included remdesivir except for sotrovimab and tixagevimab/cilgavimab; Any sotrovimab + any tixagevimab/cilgavimab: Includes all antiviral treatment which included sotrovimab or tixagevimab/cilgavimab; Plasma only + molnupiravir only: Includes single therapy with convalescent plasma or molnupiravir

**eTable 2.** Characteristics and outcome data for 593 hospitalized and non-hospitalized patients.

|  | **Overall** *(n=593)* | | **Home** *(n=284)* | | **Hospital** (n=309) | | | | **p value** |
| --- | --- | --- | --- | --- | --- | --- | --- | --- | --- |
|  |  |  |  |  | ***Overall***  (n=309) | | ***Critical infection*** (n=53) | |  |
|  | **n** | **%** | **n** | **%** | **n** | **%** | **n** | **%** | *Home vs Hospital* |
| **Sex** |  |  |  |  |  |  |  |  |  |
| Female | 258 | 43.5% | 124 | 48.1% | 134 | 51.9% | 23 | 17.2% | 0.942 |
| Male | 335 | 56.5% | 160 | 47.8% | 175 | 52.2% | 30 | 17.1% |  |
| **Age***, (IQR) [absolute range]* | 66 (54-75) [18-97] | | 64 (51-74) [20-94] | | 68 (59-77) [18-97] | | 65 (54-71) [29-88] | | <0.001 |
| 18-25 years old | 20 | 3.4% | 14 | 70.0% | 6 | 30.0% | 0 | 0.0% | 0.002 |
| 26-50 years old | 93 | 15.7% | 56 | 60.2% | 37 | 39.8% | 6 | 16.2% |  |
| 51-69 years old | 236 | 39.8% | 115 | 48.7% | 121 | 51.3% | 28 | 23.1% |  |
| ≥ 70 years old | 244 | 41.1% | 99 | 40.6% | 145 | 59.4% | 19 | 13.1% |  |
| **Comorbidities before COVID-19**^a^ |  |  |  |  |  |  |  |  |  |
| No comorbidities | 234 | 39.5% | 132 | 56.4% | 102 | 43.6% | 16 | 15.7% | <0.001 |
| 1 comorbidity | 199 | 33.6% | 95 | 47.7% | 104 | 52.3% | 19 | 18.3% |  |
| 2 comorbidities | 99 | 16.7% | 34 | 34.3% | 65 | 65.7% | 11 | 16.9% |  |
| 3 or more comorbidities | 61 | 10.3% | 23 | 37.7% | 38 | 62.3% | 7 | 18.4% |  |
| *Chronic cardiopathy* | 247 | 41.7% | 101 | 40.9% | 146 | 59.1% | 22 | 15.1% | 0.004 |
| *Chronic pulmonary disease* | 67 | 11.3% | 28 | 41.8% | 39 | 58.2% | 12 | 30.8% | 0.288 |
| *Diabetes mellitus* | 66 | 11.1% | 24 | 36.4% | 42 | 63.6% | 9 | 21.4% | 0.047 |
| *Liver disease* | 20 | 3.4% | 9 | 45.0% | 11 | 55.0% | 3 | 27.3% | 0.792 |
| *Obesity* | 41 | 6.9% | 16 | 39.0% | 25 | 61.0% | 5 | 20.0% | 0.239 |
| *Renal impairment* | 34 | 5.7% | 11 | 32.4% | 23 | 67.6% | 3 | 13.0% | 0.062 |
| *Smoking history* | 66 | 11.1% | 31 | 47.0% | 35 | 53.0% | 5 | 14.3% | 0.874 |
| *No risk factor identified* | 232 | 39.1% | 131 | 56.5% | 101 | 43.5% | 15 | 14.9% | <0.001 |
| **Baseline malignancy** |  |  |  |  |  |  |  |  | 0.620 |
| Lymphoproliferative malignancies | 417 | 70.3% | 195 | 46.8% | 222 | 53.2% | 34 | 15.3% |  |
| *Acute lymphoid leukemia* | 31 | 5.2% | 17 | 54.8% | 14 | 45.2% | 1 | 7.1% |  |
| *Chronic lymphoid leukemia* | 61 | 10.3% | 30 | 49.2% | 31 | 50.8% | 3 | 9.7% |  |
| *Hodgkin lymphoma* | 23 | 3.9% | 15 | 65.2% | 8 | 34.8% | 1 | 12.5% |  |
| *Non-Hodgkin lymphoma* | 205 | 34.6% | 85 | 41.5% | 120 | 58.5% | 17 | 14.2% |  |
| *Multiple myeloma* | 97 | 16.4% | 48 | 49.5% | 49 | 50.5% | 12 | 24.5% |  |
| Myeloproliferative malignancies | 154 | 26.0% | 74 | 48.1% | 80 | 51.9% | 18 | 22.5% |  |
| *Acute myeloid leukemia* | 66 | 11.1% | 26 | 39.4% | 40 | 60.6% | 8 | 20.0% |  |
| *Chronic myeloid leukemia* | 23 | 3.9% | 20 | 87.0% | 3 | 13.0% | 0 | 0.0% |  |
| *Myelodysplastic syndrome* | 42 | 7.1% | 14 | 33.3% | 28 | 66.7% | 5 | 17.9% |  |
| *Myelofibrosis* | 23 | 3.9% | 14 | 60.9% | 9 | 39.1% | 5 | 55.6% |  |
| Other malignancies | 22 | 3.7% | 15 | 68.2% | 7 | 31.8% | 1 | 14.3% |  |
| **Status of malignancy at COVID-19 onset**^b^ |  |  |  |  |  |  |  |  | <0.001 |
| Controlled malignancy | 320 | 54.0% | 187 | 58.4% | 133 | 41.6% | 21 | 15.8% |  |
| Stable malignancy | 104 | 17.5% | 45 | 43.3% | 59 | 56.7% | 9 | 15.3% |  |
| Active malignancy | 168 | 28.3% | 52 | 31.0% | 116 | 69.0% | 23 | 19.8% |  |
| **Malignancy treatment before COVID-19** | 548 | 92.4% | 267 | 48.7% | 281 | 51.3% | 50 | 17.8% | 0.157 |
| **Previous administration of anti-CD20 before COVID-19** | 208 | 35.1% | 85 | 40.9% | 123 | 59.1% | 18 | 14.6% | 0.012 |
| **Neutropenia^c^** | 38 | 6.4% | 11 | 28.9% | 27 | 71.1% | 5 | 18.5% | 0.048 |
| **Lymphopenia^d^** | 48 | 8.1% | 8 | 16.7% | 40 | 83.3% | 12 | 30.0% | <0.001 |
| **Previous SARS-CoV-2 vaccination** | 493 | 83.1% | 245 | 49.7% | 248 | 50.3% | 37 | 14.9% | 0.051 |
| Not vaccinated | 100 | 16.9% | 39 | 39.0% | 61 | 61.0% | 16 | 26.2% | 0.030 |
| One dose | 27 | 4.6% | 11 | 40.7% | 16 | 59.3% | 1 | 6.3% |  |
| >One dose | 466 | 78.6% | 234 | 50.2% | 232 | 49.8% | 36 | 15.5% |  |
| **COVID-19** **treatment^e^** |  |  |  |  |  |  |  |  | <0.001 |
| No treatment | 376 | 63.4% | 224 | 59.6% | 152 | 40.4% | 28 | 18.4% |  |
| Any remdesivir, minus sotrovimab | 80 | 13.5% | 7 | 8.8% | 73 | 91.3% | 8 | 11.0% |  |
| Any sotrovimab + any tixagevimab/cilgavimab | 114 | 19.2% | 40 | 35.1% | 74 | 64.9% | 14 | 18.9% |  |
| Plasma only + molnupiravir only | 22 | 3.7% | 12 | 54.5% | 10 | 45.5% | 3 | 30.0% |  |
| **Stay during COVID**-19 |  |  |  |  |  |  |  |  |  |
| Duration of the stay in hospital*, (IQR) [absolute range]* | 10 (6-19) [1-135] | |  | | 10 (6-18) [1-135] | | 17 (10-30) [1-60] | | - |
| Intermediate care | 16 | 2.7% |  |  | 16 | 100.0% | 16 | 100.0% | - |
| *Duration of the stay in intermediate care, (IQR) [absolute range]* | 10 (5-21) [1-44] | |  | | 10 (5-24) [1-44] | | 10 (5-24) [1-44] | | - |
| ICU | 44 | 7.4% |  |  | 44 | 100.0% | 44 | 100.0% | - |
| *Duration of the stay in ICU, (IQR) [absolute range]* | 12 (6-15.5) [1-30] | |  | | 11 (6-15) [1-30] | | 11 (6-15) [1-30] | | - |
| *Invasive mechanical ventilation* | 18 | 3.0% |  |  | 18 | 100.0% | 18 | 100.0% | - |
| *Non-invasive mechanical ventilation* | 18 | 3.0% |  |  | 18 | 100.0% | 18 | 100.0% | - |
| **Outcome** |  |  |  |  |  |  |  |  |  |
| *Observation time, (IQR) [absolute range]* | 23 (12-30) [0-30] | | 23 (10.5-30) [0-30] | | 23 (12-30) [0-30] | | 24 (16-30) [2-30] | | 0.375 |
| Mortality | 54 | 9.1% | 3 | 5.6% | 51 | 94.4% | 20 | 39.2% | <0.001 |
| *Reason for death* |  |  |  |  |  |  |  |  |  |
| COVID-19 | 32 | 5.4% | 1 | 3.1% | 31 | 96.9% | 13 | 41.9% | 0.199 |
| COVID-19 + hematological malignancy | 19 | 3.2% | 1 | 5.3% | 18 | 94.7% | 6 | 33.3% |  |
| Hematological malignancies +/- other reasons | 3 | 0.5% | 1 | 33.3% | 2 | 66.7% | 1 | 50.0% |  |

COVID-19, coronavirus disease 2019; ICU, intensive care unit; IQR, interquartile range; SARS-CoV-2, severe acute respiratory syndrome coronavirus 2

^a^Includes chronic cardiopathy, chronic pulmonary disease, liver disease, diabetes mellitus, obesity (BMI >30, renal impairment (creatinine >2 mg/dl), and history of smoking

^b^Controlled malignancy: Complete remission or partial remission; active malignancy: onset or refractory/resistant

^c^Absolute neutrophil count <500 cells/mm3

^d^<200 cells/mm3

^e^No treatment: Includes treatment with acyclovir, favipiravir, casivirimab/imdevimab, bamlanvimab/etesivimab and regdanvimab; Any remdesivir, minus sotrovimab: Includes all antiviral treatment which included remdesivir except for sotrovimab and tixagevimab/cilgavimab; Any sotrovimab + any tixagevimab/cilgavimab: Includes all antiviral treatment which included sotrovimab or tixagevimab/cilgavimab; Plasma only + molnupiravir only: Includes single therapy with convalescent plasma or molnupiravir

**eTable 3.** Risk factor analysis for progression to critical infection in hospitalized patients.

|  | Become ***Critical*** | | | | | | | |
| --- | --- | --- | --- | --- | --- | --- | --- | --- |
|  | **Univariable** | | | | **Multivariable** | | | |
|  | **p value** | **HR** | **95% CI** | | **p value** | **HR** | **95% CI** | |
|  |  |  | **Lower** | **Upper** |  |  | **Lower** | **Upper** |
| **Sex** |  |  |  |  |  |  |  |  |
| Female | - | - | - | - |  |  |  |  |
| Male | 0.996 | 0.999 | 0.550 | 1.814 |  |  |  |  |
| **Age** | 0.183 | 0.987 | 0.968 | 1.006 |  |  |  |  |
| **Malignancy status at COVID-19 diagnosis** |  |  |  |  |  |  |  |  |
| Controlled disease | - | - | - | - |  |  |  |  |
| Stable disease | 0.925 | 0.960 | 0.411 | 2.244 |  |  |  |  |
| Active disease | 0.405 | 1.319 | 0.687 | 2.533 |  |  |  |  |
| **Baseline malignancy** |  |  |  |  |  |  |  |  |
| Lymphoproliferative malignancies | - | - | - | - |  |  |  |  |
| Myelopolifrerative malignancies | 0.126 | 1.633 | 0.872 | 3.061 |  |  |  |  |
| Aplastic anemia | 1.000 | 0.000 | 0.000 | . |  |  |  |  |
| **Vaccination before COVID-19** |  |  |  |  |  |  |  |  |
| No vaccination | - | - | - | - | - | - | - | - |
| One dose | 0.119 | 0.188 | 0.023 | 1.536 | 0.124 | 0.188 | 0.022 | 1.581 |
| Two doses | 0.770 | 0.893 | 0.417 | 1.910 | 0.457 | 0.734 | 0.325 | 1.659 |
| Three doses | 0.006 | 0.338 | 0.157 | 0.731 | 0.003 | 0.286 | 0.127 | 0.644 |
| **Chronic cardiopathy** | 0.359 | 0.755 | 0.415 | 1.375 |  |  |  |  |
| **Chronic pulmonary disease** | 0.019 | 2.482 | 1.165 | 5.291 | 0.005 | 3.233 | 1.424 | 7.338 |
| **Diabetes mellitus** | 0.431 | 1.382 | 0.618 | 3.091 |  |  |  |  |
| **Liver disease** | 0.372 | 1.860 | 0.477 | 7.256 |  |  |  |  |
| **Obesity** | 0.694 | 1.229 | 0.440 | 3.436 |  |  |  |  |
| **Renal impairment** | 0.589 | 0.708 | 0.203 | 2.474 |  |  |  |  |
| **Smoking history** | 0.634 | 0.785 | 0.290 | 2.126 |  |  |  |  |
| **No risk factor** | 0.455 | 0.780 | 0.407 | 1.497 |  |  |  |  |
| **Lymphocytes** |  |  |  |  |  |  |  |  |
| < 200 | - | - | - | - | - | - | - | - |
| 201 - 499 | 0.119 | 0.467 | 0.179 | 1.217 | 0.108 | 0.439 | 0.160 | 1.199 |
| > 499 | 0.025 | 0.408 | 0.186 | 0.892 | 0.027 | 0.398 | 0.175 | 0.903 |

95 % CI, 95% confidence interval; COVID-19, coronavirus disease 2019; HR, hazard ratio; OR, odds ratio; SARS-CoV-2, severe acute respiratory syndrome coronavirus 2

^a^Controlled disease: Complete remission or partial remission; active disease: onset or refractory/resistant

^b^BMI >30

^c^Creatinine >2 mg/dl

**eTable 4.** Full EPICOVIDHEA questionnaire

**Identification**

Please provide details to the following questions.

Dear researcher,

Thank you very much for participating in EPICOVIDEHA. Before starting the documentation of your patients, please, let us remind you the inclusion criteria:

- Patients over 18 years of age

- Hematological malignancies (no disorders, no solid tumors. Notice, the patient can have hematological malignancies and additionally hematological disorders and/or solid tumors):

* Active, at least, during the 5 years prior to COVID-19 (diagnosis, treatment...)

* Hematological diagnosis prior to COVID-19

- Laboratory diagnosis for COVID-19 (not clinical diagnosis)

- **Principal investigator at your institution** *(Please provide name and email)*
- **Please, provide the name of your institution/hospital**
- **City**
- **Country** *(Please select the country in English)*
- **Documentation of this case in any other registries?**
  - No
  - Yes. Please specify (e.g., ECMM Aspergillus in COVID-19, LEOSS...)
- **Case already published?**
  - No
  - Yes. Please specify (digital object identifier - DOI):
- **Date of the survey** *(Day. Month. Year)***Demographics**

Please provide details to the following questions.

- **Sex**
  - Female
  - Male
- **Age**
- **Date of birth** *(Day. Month. Year)*
- **Ethnic origin** *(If the ethnic origin is unclear, please provide further details)*
- **Date of COVID-19 diagnosis** *(Day. Month. Year)*
- **Is this the first time the patient is diagnosed with COVID-19?**
  - Yes, first diagnosis
  - No, re-activation (please indicate the number of episode):
- **Did you document this patients' prior infection?**
  - No
  - Yes. Please provide the given code:
- **Type of CoV-2 infection**
  - Wild type
  - α mutation (Alpha)
  - β mutation (Beta)
  - γ mutation (Gamma)
  - δ mutation (Delta)
  - δ + mutation (Delta plus)
  - ο mutation (Omicron)
  - Other mutation/Further details. Please specify:
  - Not tested
- **Did the patient receive a vaccine against SARS-CoV-2?**
  - Yes
  - No, vaccination was denied by:
  - No, unknown reasons
  - Unknown whether vaccinated
- **Where did the patient stay during the COVID-19 infection?**
  - Admitted in hospital
  - At home
  - Other. Please specify

**SARS-CoV-2 vaccination**

You stated the patient received SARS-CoV-2 vaccine. Please provide further details.

| **Dose** | **Vaccine name** | **Vaccine administration date** | **Serology? (y/n)** | **Serology sample date** | **Anti-S IgG** | | | **Neutralizing ABs** | | |
| --- | --- | --- | --- | --- | --- | --- | --- | --- | --- | --- |
|  |  |  |  |  | **Level** | **Test** | **Cut-off** | **Level** | **Test** | **Cut-off** |
| #1 |  |  |  |  |  |  |  |  |  |  |
| #2 |  |  |  |  |  |  |  |  |  |  |
| #3 |  |  |  |  |  |  |  |  |  |  |
| #4 |  |  |  |  |  |  |  |  |  |  |
| #5 |  |  |  |  |  |  |  |  |  |  |

- **If you want to provide further details, please use the space below.**

**Underlying diseases**

- **Which comorbidities were present before the COVID-19 infection?**
  - Chronic cardiopathy (atrial fibrillation, hypertension, obstructive arteriopathy...)
  - Chronic pulmonary disease (asthma, COPD, cystic fibrosis, fibrosis...)
  - Diabetes (treated with insulin or antidiabetic oral drugs)
  - Liver disease
  - Obesity (BMI >30) or Underweight (BMI <18.5), please indicate BMI:
  - Renal impairment (Creatinine> 2mg/dl)
  - Smoker, please indicate pack years:
  - Other risk factors (including bacterial, fungal and/or viral infections). Please specify:
  - No risk factor identified
- **Please provide the following details at diagnosis of COVID-19**
  - Absolute Leukocyte Number *(units/mm³)*
  - Absolute Neutrophil Number *(units/mm³)*
  - Absolute Lymphocyte Number *(units/mm³)*
- **If you want to provide further details, please use the space below.**

**Hematological malignancy**

Please provide further details on the condition of the patient.

- **Type of malignancy**
  - Leukemia
    - Acute lymphoblastic leukemia
    - Chronic lymphocytic leukemia
    - Acute myeloid leukemia
    - Chronic myeloid leukemia
  - Lymphoma
    - Hodgkin lymphoma
    - Non-Hodgkin lymphoma
  - Other
    - Amyloid light-chain amyloidosis
    - Aplastic anemia
    - Essential thrombocythemia
    - Hairy cell leukemia
    - Multiple myeloma
    - Myelodysplastic syndrome
    - Myelofibrosis
    - Polycythemia vera
    - Systemic mastocytosis
    - Other. Please specify
- **Details on the diagnosis**
- **State of hematological malignancy at the collection day of COVID-19 diagnosis**
  - Onset
  - Complete remission
  - Partial remission
  - Stable disease
  - Refractory/Resistant
  - Unknown
- **How many months prior to the COVID-19 diagnosis was the above reported diagnosis made?** *(0 if same month)*
- **Was there a simultaneous diagnosis of COVID-19 and hematological malignancy?** *(Time span of 1 month or less between hematological malignancy diagnosis and COVID-19 diagnosis)*
  - Yes
  - No
- **Type of treatment**
  - Chemotherapy *(including antineoplastics, immunosuppressives, corticosteroids, antibodies and/or small molecules)*
  - Radiotherapy
  - Hematopoietic Stem Cell Transplantation (HSCT) - Allogeneic
  - Hematopoietic Stem Cell Transplantation (HSCT) - Autologous
  - CAR-T
  - Other. Please specify
  - No treatment

**Simultaneous malignancy and COVID-19 diagnosis**

In the previous page you stated that there was a simultaneous malignancy and COVID-19 infection diagnosis. Please provide further details

- **Date of hematological malignancy diagnosis** *(Day. Month. Year)*
- **Was the treatment for hematological malignancy immediately needed?**
  - Yes, before COVID-19 diagnosis
  - Yes, after COVID-19 diagnosis
  - No
- **Was the treatment for hematological malignancy delivered?**
  - Yes
  - No

**If the patient received treatment for the hematological malignancy...Please, provide full details of the treatment**

- **Date of hematological malignancy treatment start** *(Day. Month. Year)*
- **Please, state how many chemotherapy courses (cycles) were administered in the first chemotherapy line.**
- **Was there a chemotherapy dose reduction?**
  - Yes. Please provide details:
  - No
- **State of the hematological malignancy treatment response after first line of treatment**
  - Complete remission
  - Partial remission
  - Stable disease
  - Refractory/Resistant
  - Unknown but active
  - Unknown but in remission
  - Unknown
- **Was there a chemotherapy induced neutropenia?** *Level of neutropenia (<500) during the acute phase of COVID-19*
  - Yes
  - No
- **Was there a secondary infection after hematological malignancy treatment administration?**
  - Yes. Please provide details:
  - No
- **Status of hematological malignancy at last follow up**
  - Ongoing
  - Improved
  - Resolved
  - Unknown
- **If there was a hematological malignancy relapse after COVID-19 infection, please provide the date of the first relapse** *(Day. Month. Year)*

**Chemotherapy**

- **Last/ongoing chemotherapy treatment**
  - Induction
  - Consolidation
  - Reinduction
  - Maintenance
  - Palliative
  - Preparation regimen
- **When was last line of chemotherapy given to the patient?**
  - In the last month
  - In the last 3 months
  - Chemotherapy ended > 3 months before COVID-19
- **Number of lines**
- **Antineoplastic drugs**
  - No
  - Unknown
  - Yes. Please select which

| Bendamustine | Carboplatin | Cyclophosphamide | Doxorubicin | Idarubicin | Mitoxantrone | Other |
| --- | --- | --- | --- | --- | --- | --- |
| Bleomycin | Cisplatin | Cytarabine | Etoposide | Melphalan | Pentostatin |  |
| Busulfan | Cladribine | Daunorubicin | Fludarabine | Methotrexate | Vincristine |  |

- **Immunosuppressive drugs**
  - No
  - Unknown
  - Yes. Please select which

| Azathioprine | Mercaptopurine | Sirolimus | Other |
| --- | --- | --- | --- |
| Cyclophosphamide | Methotrexate | Tacrolimus |  |
| Cyclosporine (CSA) | Mycophenolate mofetil (MMF) | Temsirolimus |  |

- **Corticosteroids**
  - No
  - Unknown
  - Yes
- **Antibodies**
  - No
  - Unknown
  - Yes. Please select which

| Alemtuzumab | Brentuximab | Gemtuzumab-Ozagamicin | Ofatumumab | Tocilizumab |
| --- | --- | --- | --- | --- |
| Anti-Thymocyte Globulin (ATG) | Daclizumab | Infliximab | Pembrolizumab | Other |
| Bevacizumab | Eculizumab | Nivolumab | Rituximab |  |

- **Small molecules**
  - No
  - Unknown
  - Yes. Please select which

| Bortezomib | Everolimus | Imatinib | Midostaurin | Ruxolitinib | Other |
| --- | --- | --- | --- | --- | --- |
| Bosutinib | Ibrutinib | Ivosidenib | Nilotinib | Sorafenib |  |
| Dasatinib | Idelalisib | Lenalidomide | Pomalidomide | Thalidomide |  |

**Allogeneic HSCT**

You stated the patient received an allogeneic stem cell transplantation. Please provide further details.

- **Allogeneic HSCT - Type of transplant**
  - Peripheral stem cells
  - Bone marrow
  - Cord blood
  - Other. Please specify:
  - Unknown
- **Time span between allogeneic HSCT and the COVID-19 diagnosis** *(days)*
- **Time span between allogeneic HSCT and neutrophil engraftment** *(days)*
- **State of hematological malignancy at the day of allogeneic HSCT**
  - Onset
  - Complete remission
  - Partial remission
  - Stable disease
  - Refractory/Resistant
  - Unknown
- **HCT-comorbidity index:**
- **Was this a myeloablative transplantation?**
  - Yes
  - No
  - Reduced intensity conditioning
  - Unknown
- **HLA matching**
  - Un-/related
  - Mismatches
- **CRS and ICANS grades** *(ASTCT consensus)*
  - CRS
  - ICANS
- **Did the patient receive immunosuppressive or corticosteroids within the last 6 months prior to COVID-19?**
  - Immunosuppressives
  - Corticosteroids
  - None
- **Did the patient receive prophylaxis towards GvHD?** *(Please, specify)*
- **If the patient developed GvHD please provide further details**
  - Type of GvHD
    - Acute
      - Degree before COVID-19 diagnosis
      - Degree at COVID-19 diagnosis
    - Chronic
      - Degree before COVID-19 diagnosis
      - Degree at COVID-19 diagnosis
    - Unknown
    - Not applicable
- **If you want to provide further details, please use the space below.**

**Autologous HSCT**

You stated the patient received an autologous stem cell transplantation. Please provide further details.

- **Type of transplant**
  - Peripheral stem cells
  - Bone marrow
  - Other. Please specify:
  - Unknown
- **Time span between autologous HSCT and the COVID-19 diagnosis** *(days)*

**CAR-T**

You stated the patient received CAR-T. Please provide further details.

- **Did the patient receive bridging therapy?**
  - Yes
  - No
  - Unknown
- **Which lymphodepletion did the patient receive for CAR-T?**
  - Bendamustine
  - Fludarabine-Cyclophosphamide
  - Other. Please elaborate:
  - Not applicable
  - Unknown
- **Please provide data on the CAR-T construct**
  - Axicabtagene ciloleucel (Axi-cell)
  - Lisocabtagene maraleucel (Liso-cell)
  - Tisagenlecleucel (Tisa-cell)
  - Other. Please elaborate:
  - Not applicable
  - Unknown
- **Time span between CAR-T and the COVID-19 diagnosis *(****days)*
- **CRS and ICANS grades** *(ASTCT consensus)*
  - CRS
  - ICANS
- **Was the patient admitted in an ICU after CAR-T due to complications?**
  - Yes
  - No
  - Unknown
- **Did the patient receive tocilizumab due to CAR-T?**
  - Yes
  - No
  - Unknown
- **Did the patient receive corticosteroids due to CAR-T?**
  - Yes
  - No
  - Unknown

**COVID-19 infection**

- **How was performed the identification of COVID-19?**
  - Bronchoalveolar lavage positive for SARS-CoV-2
  - Nasopharyngeal swab positive for SARS-CoV-2
  - SARS-CoV-2 serology
  - Other. Please specify
- **Why was performed COVID-19 test?**
  - Extrapulmonary symptoms *(anosmia, fever, skin signs...)*
  - Pulmonary symptoms *(cough, dyspnea, imaging signs...)*
  - Screening
  - Other. Please specify
- **Was the patient admitted into ICU during COVID-19 infection?**
  - Yes
  - No
  - Unknown
- **Please, provide details on the ICU stay of the patient as applicable:**
  - Ventilation
    - Invasive mechanical ventilation
    - Non-invasive mechanical ventilation
    - Unknown
  - Inotropic support
  - Transfusions
  - Anticoagulant administration

**Antiviral treatment of COVID-19**

- Was antiviral treatment administered to this patient? *(Antiviral treatment including antivirals drugs, convalescent plasma and fluvoxamine)*
  - Yes (Please, provide details below)
  - No
  - Unknown
  - Please, state which antiviral/antibody treatment were administered to this patient against COVID-19?

| **Antiviral** | **Drug name** | **Start date** | **Stop date** |
| --- | --- | --- | --- |
| #1 |  |  |  |
| #2 |  |  |  |
| #3 |  |  |  |
| #4 |  |  |  |
| #5 |  |  |  |

- - Was serology performed before start of COVID-19 treatment?
    - Yes. Please state date, result and test name:
    - No
    - Unknown
  - If treatment specific antiviral treatment were used...Was sequencing performed BEFORE start of treatment?
    - Yes *(Please, provide details below)*
    - No
    - Unknown
  - If treatment specific antiviral treatment were used...Was sequencing performed AFTER start of treatment?
    - Yes *(Please, provide details below)*
    - No
    - Unknown
  - Please, provide details if sequencing BEFORE AND/OR AFTER treatment was performed, including whether there were any mutations associated with antiviral resistance found
- **Please, provide additional details to the treatment stated above or, alternatively, a summary of the drugs administered to the patient in order to treat COVID-19 if they do not fit in the questions above** *(corticosteroids, experimental therapy...)*

**Outcome**

- **Was the patient alive at last contact?**
  - Yes
  - No
- **Which was the last date of follow up?** *Last day of follow up is the last day of contact with the patient. (Day. Month. Year)*
- **Duration of the inpatient total stay** *(days)*
  - How many days was the patient on the following wards?
    - Normal ward *(days)*
    - Intermediate care *(days)*
    - Intensive care unit *(days)*
    - COVID-19/Other ward *(days)*

**Patient died**

You stated that the patient died. Please provide further details.

- **Date of death** *(Day. Month. Year)*
- **Was the death attributable to:**
  - Attributable to COVID-19
  - Contributable by COVID-19
  - Non-related to COVID-19
  - Attributable to hematological malignancy
  - Attributable to unknown reasons
  - Attributable to other reasons. Please state below:
- **Primary cause(s) of death:**
